# Rethinking grapevine downy mildew management: opportunities to disrupt the sexual cycle of the pathogen as a preventive disease control strategy

**DOI:** 10.3389/fpls.2026.1843162

**Published:** 2026-05-20

**Authors:** Paige Breen, Charlotte Poeydebat, François Delmotte

**Affiliations:** Vineyard Health and Agroecology (SAVE), National Research Institute for Agriculture, Food and Environment (INRAE), Bordeaux Sciences Agro, Institute of Vine and Wine Sciences (ISVV), Villenave d’Ornon, France

**Keywords:** epidemiology, fungal pathogens, fungal sexual reproduction, grapevine, oomycete, plant disease, *Plasmopara viticola*, viticulture

## Abstract

Plant diseases caused by fungal and oomycete pathogens with mixed reproduction systems include some of the most damaging crop diseases. These pathogens reproduce asexually under low-stress conditions during the growing season in order to propagate rapidly and efficiently. They then shift to sexual reproduction when conditions become harsher, so as to survive and adapt. For those that are obligate biotroph pathogens with a deciduous host, sexual reproduction is a necessary step in their life cycle. *Plasmopara viticola*, the oomycete causing grapevine downy mildew (GDM), is a representative example, particularly in temperate regions in which grapevines enter dormancy in autumn. Current efforts to control GDM, including those comprising Integrated Pest Management (IPM), are typically limited to the grapevine’s growing season. They target the primary contamination and the asexual propagation of *P. viticola* and consist most typically of repeated fungicide applications whenever contamination conditions are met. However, strategies that would target the sexual cycle of *P. viticola* during the offseason are often overlooked, even though related approaches are successful in other plant-pathogen systems. We discuss several novel interventions informed by plant pathogen biology, epidemiology, and ecology that would disrupt the pathogen’s sexual cycle at several key points, reducing both the epidemic pressure and the ability of the pathogen to adapt from one growing season to the next. Furthermore, we identify the main scientific and technical challenges to be overcome. In line with IPM, we call for a shift toward a preventive, long-term vision of plant disease management and more sustainable agroecosystems.

## Introduction

Fungal and oomycete pathogens are responsible for some of the most damaging plant diseases, leading to substantial crop loss and threatening global food security (Fisher et al., 2012; Bebber and Gurr, 2014; Steinberg and Gurr, 2020). Among these pathogens, the most successful have mixed reproduction systems and retain the ability to reproduce sexually (McDonald and Linde, 2002; Heitman, 2006), granting them several advantages and making them particularly difficult to control. For example, they benefit from the rapid, clonal and exponential multiplication and propagation enabled by asexual reproduction, made even easier in agroecosystems where host stress is systematically minimized by agricultural practices. In addition, the genetic recombination and consequent evolutionary potential of undergoing at least one sexual cycle per year (McDonald and Linde, 2002) enables them to more quickly adapt to environmental changes, resistant host plants, fungicides, and other efforts to control them (Lamour and Hausbeck, 2000; Lamour and Hausbeck, 2001; Clément et al., 2010). Moreover, the sexually-produced form survives adverse conditions (*e.g.*, lack of vulnerable host plant tissue and drought) and enables the pathogen to start new epidemics in the following season and even years later (van den Berg et al., 2001; Babadoost and Pavon, 2013; Wyatt et al., 2013). For some of these pathogens (namely, obligate biotrophs with an annual or perennial but deciduous host), their survival from one season to the next depends on their ability to form conservation structures such as oospores. This means sexual reproduction is an essential–and therefore vulnerable–step in their life cycle.

**Figure 1:**
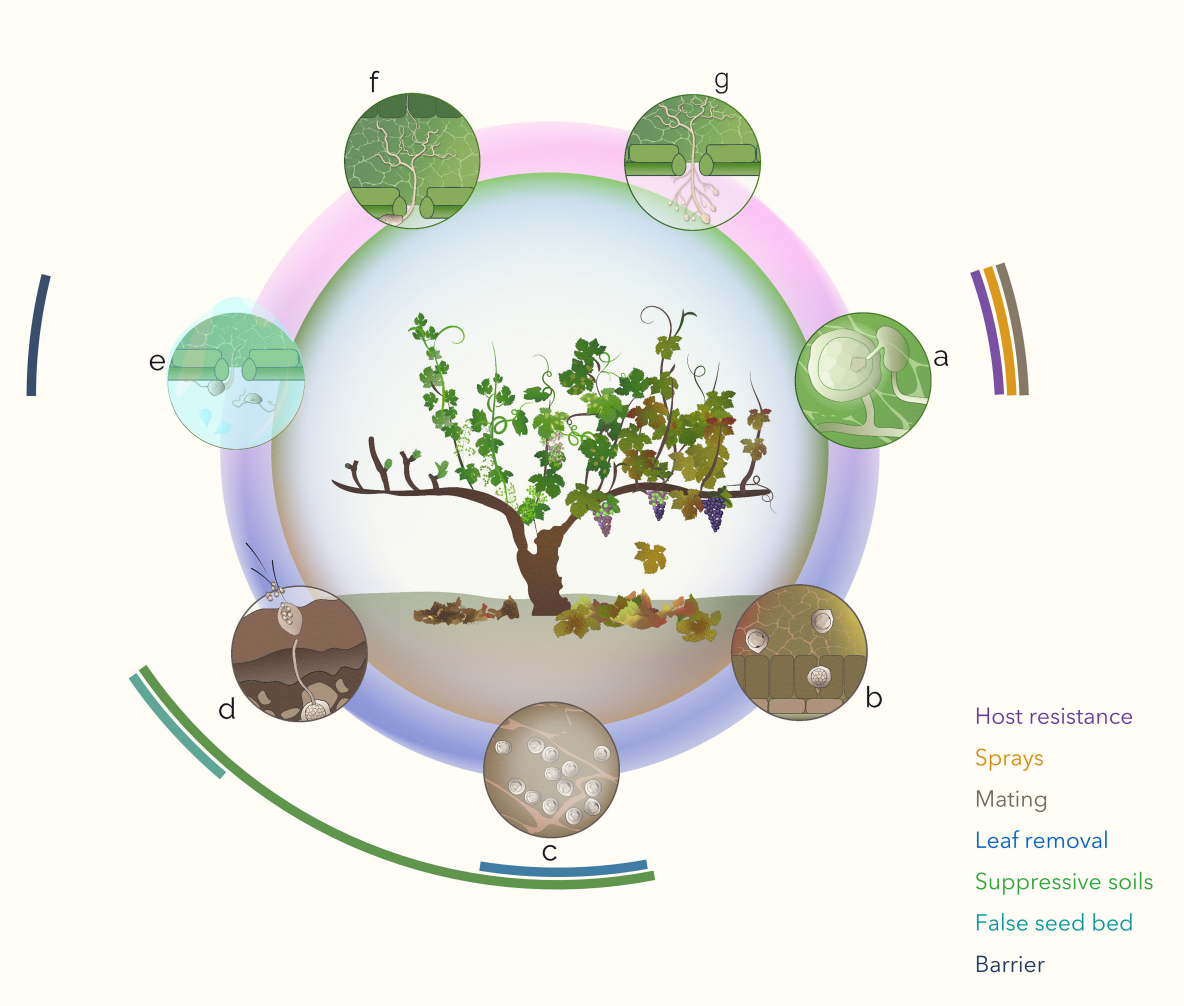
Representation of the key interventions proposed at different steps of *Plasmopara viticola*’s sexual (dark blue inner arc) cycle: **a)** heterothallic sexual reproduction, or fertilization of oogonia by antheridia in the leaf mesophyll of an infected area starting when stress conditions are met; **b)** oospore formation involving nuclear fusion and thickening of the inner oosphere wall in the leaf mesophyll; **c)** oospore maturation and dormancy in leaf litter and the surrounding soil, which then comprises the primary inoculum reservoir for the next year; **d)** germination, including mitotic division of the nucleus and the eventual release of uninucleate, motile, biflagellate zoospores; **e)** primary contamination of the host plant, comprised of zoospore-containing water droplets reaching vulnerable parts of the plant and then encystment on abaxial face of leaf, elongation of germination tube and penetration through stomata; **f)** mycelial growth and extraction of nutrients from the host plant; and **g)** asexual production of sporangiophores that then release zoospores, which travel by rain and wind to cause secondary contaminations. Outer arcs illustrate which step(s) of the cycle each intervention targets (detailed in **Table 1**).

*Plasmopara viticola* (Berk. & M.A. Curtis) Berl. & De Toni, the causal agent of grapevine downy mildew (GDM), is representative of this kind of pathogen, particularly in temperate regions where grapevines (*i.e.*, *Vitis vinifera*) enter dormancy in autumn. *P. viticola*’s reproductive cycle is comprised of two parts: the asexual cycle, which occurs during the grapevine’s growing season and in which the zoospores originating from lesions propagate on green parts of the vine; and the sexual cycle, in which the crossing of strains in the leaves towards the end of the grapevine growing season leads to the formation of oospores that overwinter in grapevine leaf debris and the surrounding soil. The key steps of the pathogen’s life cycle are represented in **Figure 1** (Galbiati, 1980; Galbiati and Longhin, 1984; Vercesi et al., 1999; Burruano, 2000; Gessler et al., 2011). While it was traditionally understood that epidemics were triggered by only a few primary contamination events followed by asexual propagation throughout the season (Lafon and Clerjeau, 1988), recent technological advances and the development of molecular markers have demonstrated that primary inocula (*i.e.*, oospores) play an important role in epidemics throughout the growing season and well beyond grapevine flowering, particularly under certain environmental conditions (Stark-Urnau et al., 2000; Gessler et al., 2003; Rumbou and Gessler, 2004; Gobbin et al., 2006; Koopman et al., 2007; Rumbou and Gessler, 2007; Yin et al., 2014; Li et al., 2016). More specifically, one study showed that at the field level, approximately 70% of identified *P. viticola* genotypes appeared only once (Koopman et al., 2007), while another indicated that up to 40% of contaminations were caused by primary inocula well beyond grapevine flowering when climate conditions are met (Hong et al., 2020). In addition, Fontaine et al. (2013) demonstrated that European *P. viticola* populations have a panmictic genetic structure characterized by random mating and a lack of significant deviation from Hardy–Weinberg equilibrium, further confirming the essential role of this pathogen’s sexual cycle. Finally, greater late-season GDM occurrence (*i.e.*, mildew mosaic symptoms) was shown to be related to earlier spring onset and higher intensity in the following growing season’s epidemic (Carisse, 2016). From these findings, *P. viticola* epidemics can best be described as a multitude of unique primary contaminations, rather than the result of a few dominant genotypes propagating throughout the vineyard. Given the primary inoculum reservoir’s significant and ongoing contribution to GDM epidemics, any interventions to mitigate it have the potential to drastically reduce subsequent disease pressure.

Current GDM management strategies remain disproportionately focused on the parts of the pathogen life cycle that occur during the host plant growing season. In particular, they aim to prevent primary contaminations and asexual propagation (Corkley et al., 2022; Massi et al., 2021), key tenets of Integrated Pest Management (IPM). This approach is carried out mainly *via* repeated–and carefully timed– spray applications (fungicides and biocontrol products), but also includes the deployment of resistant varieties and the management of microclimates *via* cultural practices (Goldammer, 2018). While these techniques often result in valuable disease suppression, they require frequent interventions, constant monitoring, and may still result in significant crop damage and loss (Fisher et al., 2012). Furthermore, many conventional fungicides have a negative impact on human health and biodiversity (Fisher et al., 2012). Despite these limitations, the underlying ideas informing current management strategies and IPM in viticulture overlook the potential advantages of targeting the sexual cycle, particularly the steps that occur after harvest and during the host plant’s dormant season.

In this Perspective article, we use *P. viticola* to illustrate that disrupting the sexual cycle of pathogens for which it is a necessary step offers three compelling advantages for disease management. First, these strategies would decrease the quantity of primary inoculum and therefore lower the epidemic intensity in the following growing season(s) (Carisse, 2016). Second, they would reduce the production and survival of genetically diverse individuals, thereby limiting the adaptive potential of pathogen populations (Didelot et al., 2016). And finally, management actions targeting the sexual cycle can be easily integrated with existing practices, such as biocontrol solutions and the use of genetically resistant crop varieties, and expand upon the aims of IPM to create a more effective, integrated, sustainable, and economically viable strategy for plant disease management.

Building on recent advances in our knowledge of pathogen biology, epidemiology, and ecology, new research methodology, and successful, related approaches in other plant-pathogen systems, we propose new paths for interventions that would interfere with different steps of the sexual cycle of *P. viticola* (summarized in **Table 1**). Furthermore, we identify the main knowledge gaps to be addressed, challenges to be overcome, and critical questions for future research and development before these measures can be more widely adopted.

**Table 1:** Summary of proposed interventions.

Intervention (short)	Intervention	Method	Sexual cycle step	General comments
*Inhibit the development of the disease on the leaves*				
Host resistance	Leverage host resistance that inhibits sexual reproduction	Genetic	a	Builds upon existing IPM strategy
Sprays	Apply post-harvest sprays	Chemical or biological	a	Builds upon existing IPM strategy
*Prevent sexual reproduction in the leaves*				
Mating	Interfere with signaling that regulates mating behavior	Biochemical	a	New to this plant-pathogen system
*Reduce the primary inoculum reservoir in the leaf litter and surrounding soil*				
Leaf removal	Remove infected plant material	Physical	c	Established idea not yet optimized for this plant-pathogen system
Suppressive soils	Promote suppressive soils	Biological	c, d	Developing idea not yet optimized for this plant-pathogen system
*Prevent the primary inocula from germinating when the plant is most vulnerable*				
False seedbed	Use the false seedbed technique	Biological or physical	d	Established idea not yet optimized for this plant-pathogen system
*Prevent the pathogen from reaching vulnerable plant tissues*				
Barrier	Prevent the inocula from reaching vulnerable host plant tissues	Physical	e	Already deployed in some commercial systems; not yet fully optimized for this plant-pathogen system

This table summarizes the proposed interventions aimed at interrupting the sexual cycle of GDM, listed in the order in which they could be effective. It includes the general method, the sexual cycle step (letter corresponding to **Figure 1**) it would target, and general comments regarding its novelty.

## Interventions

### Leverage host resistance that inhibits sexual reproduction

Grapevine breeding programs produce new, GDM-resistant varieties by introgression of known resistance *loci* from wild, non-*Vitis vinifera* grapevine species. These resistance *loci* have been shown to inhibit the primary contamination and asexual propagation of *P. viticola* (**Figure 1** steps e–g) (Possamai and Wiedemann-Merdinoglu, 2022; Trapp et al., 2023; Paire et al., 2024). However, even in the presence of these *loci*, the pathogen can complete its life cycle, including sexual recombination and primary inoculum formation in host leaves (Delbac et al., 2019). The role of host plant traits in pathogen sexual reproduction has been studied in other plant-pathogen systems, such as potato late blight, in which the authors theorized that there were similar host nutrient requirements for both asexual propagation and sexual reproduction of the pathogen (Clément et al., 2010). New research methods, particularly those that quantify primary inocula in leaves using quantitative PCR, could facilitate the identification and evaluation of resistance genes specifically for their effect on pathogen sexual reproduction and oospore formation (**Figure 1** steps a, b) (Vercesi et al., 2010; Si Ammour et al., 2020; Yang et al., 2023). Expanding how GDM-resistant varieties are assessed, by taking into account more of the pathogen life cycle, and their subsequent deployment would not result in total disease control, but would rather improve the efficacy and long-term stability of these varieties. It would also help to make host resistance genes, a limited resource, more durable (McDonald and Linde, 2002). First of all, though, it is necessary to develop and establish protocols that identify grapevine traits and resistance genes with the power to inhibit the pathogen’s sexual cycle. Plus, as with conventional resistant varietal selection, the process is decades-long and must address multiple challenges, including adaptability to climate change, impeccable wine quality, and evolving consumer preferences (Santos et al., 2020; Töpfer and Trapp, 2022). Finally, while the deployment of such resistant varieties would not be any more complicated than it is for existing resistant varieties, regulations and the low rate of vineyard replanting make it a slow intervention to implement more broadly (Töpfer and Trapp, 2022).

### Apply post-harvest sprays

Normally, spray applications for GDM control aim to prevent primary contaminations and asexual propagation during the grapevine growing season (**Figure 1** steps e–g). They are typically stopped a few weeks before harvest, both because the crop is no longer vulnerable and to account for the set pre-harvest interval. However, GDM continues to propagate asexually and unchecked, particularly under often increasingly favorable autumn environmental conditions (Salinari et al., 2006). Late-season treatments aiming to address this challenge in other systems have been studied in apple trees, for example, and have been shown to be effective at ensuring the durability of resistance genes against apple scab (caused by the ascomycete *Venturia inaequalis*) (Didelot et al., 2016). Other research has shown how strategically timed treatments on the leaves, such as of the biocontrol agent *Microsphareopsis ochracea*, reduce the apple scab primary inoculum reservoir, decreasing the needed number of spraying operations during the following season (Carisse and Rolland, 2004). To target sexual reproduction (**Figure 1** step a) with post-harvest sprays, it is essential to better understand autumn disease occurrence and progress; to evaluate existing and new products in late-season environmental conditions; and to assess how these applications ultimately affect the extent of primary inoculum reservoir recharging and the intensity of the following season’s epidemic. Studies are currently underway to quantify the efficacy of late-season treatments on the dynamics of primary inoculum production, and they are expected to have a limited but meaningful mitigating effect. In addition, late-season treatments would be simple to implement, as the growers almost certainly already possess the necessary equipment.

### Interfere with signaling that regulates mating behavior

Hormonal regulation of sexual reproduction in the oomycete *Phytophthora* was first proposed by Ko (1978), which hypothesized the existence of mating-type-specific factors. This hypothesis was subsequently validated through the characterization of these hormones in the heterothallic species *Phytophthora infestans*, showing that pheromone biosynthesis is interdependent (Ojika et al., 2011), that each mating type requires the intermediate produced by the other (Ariyoshi et al., 2021), and that these hormones were to some extent shared with downy mildew oomycetes (Tomura et al., 2017). Likewise, *P. viticola* requires the presence and coming together of two compatible mating types (P1 and P2) within infected leaves in order to undergo sexual reproduction (**Figure 1** step a) (Ko, 1988; Wong et al., 2001; Scherer and Gisi, 2006). While the mating-type locus in *P. viticola* has been identified (Dussert et al., 2020), further investigation is required to characterize the candidate genes involved in mating-type determination, particularly those encoding receptors that mediate signal perception and recognition. It is also necessary to identify downy mildew-specific hormones and define the molecular pathways underlying their biosynthesis, ultimately devising a way to disrupt mating-type signaling and prevent interactions between compatible mating types (Fabritius et al., 2002; Harutyunyan et al., 2008; Judelson and Ah-Fong, 2019). If an intervention based on the use of mating-type hormones is proven to be effective at controlling GDM, much more work will be needed before it can be widely adopted in viticulture, including studies of its efficacy in the field, determining how to industrially produce the product, and undergoing the lengthy process to be registered as a biocontrol product.

### Remove infected plant material

For many annual plants, crop rotation and the removal of infected material are effective strategies to reduce the primary inoculum of diseases and lower epidemic pressure (Sosnowski et al., 2009; Boualleg et al., 2024). In perennial crops, rotation is not possible and the removal of infected plant organs is less commonly implemented. However, there have been successful studies testing the treatment (or “destruction”) of apple scab-infected leaf litter in orchards *via* leaf shredding, chemical or biocontrol treatments, or urea applications in order to reduce the next season’s disease pressure (Carisse and Rolland, 2004; Boualleg et al., 2024; Stewart et al., 2025). In the case of GDM, the disposal of leaves in autumn or the use of preventive defoliants could serve as an effective measure to reduce the primary inoculum reservoir in vineyard soils (**Figure 1** step c). Leaves that are physically removed could even be composted in order to “deactivate” GDM primary inocula *via* heating or microbial interactions before returning the organic matter to the vineyard (Courchinoux et al., 2025). While studies assessing this intervention in the field are currently underway, its efficacy is expected to be quite high, as supported by the fact that similar interventions are already well-established in many other plant-pathogen systems. However, further study is needed to better understand when and under what conditions oospores start to appear in the leaves; leaf fall dynamics; how long primary inocula survive in the soil; how leaf removal affects the primary inoculum reservoir recharging and the following season’s disease pressure; at what spatial scale the measure must be implemented in order to be effective; and any long-term detrimental effects on the soil (*i.e.*, nitrogen, carbon) and grapevine (*i.e.*, health and vigor). One other anticipated barrier to widespread adoption is that the removal of infected material, without the proper machinery, is labor-intensive. However, this machinery is currently being developed and, once commercially available, would make the removal of infected material straightforward for viticulturists to incorporate into their vineyard operations.

### Promote suppressive soils

The idea of suppressive soils, or soils unfavorable to plant disease due to their microbial composition, is typically used in relation to rhizosphere diseases. However, there is reason to believe the concept could also apply to pathogens that spend any part of their life cycle in the soil (Baker and Cook, 1974). Advances in Next-Generation Sequencing now enable researchers to identify soil microbial compositions (Schlatter et al., 2017). Such techniques have recently showed, for example, that not only is there an exchange between leaf and topsoil microbiota in vineyards and other plant-pathogen systems (in GDM and *Hyaloperonospora arabidopsidis* in *Arabidopsis thaliana*, respectively; Fournier et al., 2025; Spooren et al., 2025), but that certain microbial taxa are more present in the topsoil of low-GDM incidence vineyards (Fournier et al., 2025). Because GDM primary inocula mature, overwinter, and often germinate in the soil, it is possible that the soil microbial composition and fauna interfere at any or all of these stages (**Figure 1** steps c, d). By prioritizing soil health (*i.e.*, protecting and encouraging microbial diversity, limiting perturbations such as tilling and fungicide spraying) and amending with applications of beneficial microorganisms including pathogen antagonists, the surrounding plants could become more resilient to disease (De Vrije et al., 2001; Toffolatti et al., 2018; Fournier et al., 2025; Spooren et al., 2025). Once certain microorganisms are confirmed to interact with and inhibit the sexual cycle of the pathogen’s life cycle, particularly germination in the soil, they must be evaluated for their efficacy in field conditions and industrially produced. In addition, research is needed to better understand how additional conservation biological control efforts, plant diversification, and interactions with other vineyard micro- and macro-fauna impacts components of *P. viticola*’s sexual cycle. Finally, because this intervention may be less straightforward to implement than some of the others, training and support must be provided to winegrowers regarding relevant soil-enhancing practices. In addition, because the beneficial effects may not be immediately obvious, policies incentivizing sustainable soil management and cross-sector collaboration to scale up the most effective techniques must be enacted.

### Use the false seedbed technique

Like in many plant-pathogen systems, *P. viticola* is phenologically aligned with its host. It germinates at moments in which the grapevine is most susceptible to contamination, continuing to germinate and infect vulnerable, young plant tissues throughout almost the entire growing season (**Figure 1** step d) (Kennelly et al., 2007; Maddalena et al., 2021). The false seedbed technique, which was originally developed as a way to manage weed populations in horticulture (Riemens et al., 2007), aims to desynchronize host and pest phenologies. In apple orchards, for example, strategic irrigation of the infected leaf litter causes *Venturia inaequalis* ascospores to release at moments otherwise unsuited to host contamination, resulting in dramatically reduced disease pressure (Prodorutti et al., 2024). A similar approach could also be envisioned for GDM, and would involve triggering the primary inocula to germinate at moments in which the plant is not vulnerable to contamination (*i.e.*, before the start of grapevine budburst), thereby reducing the primary inoculum reservoir. However, to deploy this intervention, it is essential to first better understand when and under what conditions primary inocula germinate in the field (*i.e.*, in the leaf litter and the less-studied soil compartment), and then develop and test methods and tools that would force primary inoculum germination. If the false seedbed technique is shown to be a promising strategy in GDM control, significant work is needed before it could be practically implemented.

### Prevent the inocula from reaching vulnerable host plant tissues

The primary inocula that lead to GDM epidemics in the following growing season are auto-inoculum, overwintering primarily in the leaf litter and surrounding soil (Rossi and Caffi, 2007). Most grapevines are trained at least a half meter off the ground, so the pathogen must travel in water droplets and air to reach vulnerable tissues of the host plant (**Figure 1** step e). Physical barriers that could prevent such transport include the use of geotextiles, mulching, and covercropping (Kassemeyer et al., 2015; Fernandes de Oliveira et al., 2021; Hasanaliyeva et al., 2024); and primary inoculum burial (as seen in the cases of *Chara aspera* and *Phytophthora capsici*, respectively; van den Berg et al., 2001; Babadoost and Pavon, 2013). Along these lines, the timing of certain vineyard interventions, such as tillage and mowing, has been shown to correspond to peak aerial spore capture events (Asinari et al., 2026), and should therefore be kept to a minimum during periods favorable to GDM contamination. In the case of a particularly promising physical barrier–spring cover cropping–additional studies are needed to quantitatively assess its efficacy, expanding upon the results from Hasanaliyeva et al., 2024 that showed a 75-95% reduction in rain-originated droplets escaping from the ground depending on cover crop height. If proven to be an effective GDM control strategy when optimized for that purpose, this intervention could be easily implemented in the field, especially as growers seek to leverage strategic cover cropping and prioritize soil health (Wezel et al., 2009; Labarga et al., 2024). Other proposed physical barriers, such as geotextiles, mulching, and primary inoculum burial, would also be relatively simple to incorporate into existing vineyard management strategies, if effective at GDM control. To evaluate these barriers, it is necessary to develop tools that enable us to better understand and measure the dispersion pathways and timing of *P. viticola* from the soil and leaf litter to the air. In addition, any unintended consequences of these barriers must be considered, including resource competition with the grapevine, detrimental effects on the microclimate, and the possibility of creating a more favorable environment for other pathogens (Muscas et al., 2017; Abad et al., 2021).

## Outlook

The need to identify, develop, and implement innovative and sustainable approaches to plant disease management in agriculture has never been greater. Disrupting the pathogen’s sexual cycle, with a particular aim to reduce the primary inoculum reservoir and inhibit germination, is one solution. To that end, it is necessary to strategically and synergistically integrate several of the interventions proposed here into a multi-faceted, preventive management strategy. Combining these innovative interventions with those already associated with IPM in viticulture and those that mitigate secondary dispersal is essential for achieving sustainable disease control. We are calling for a paradigm shift toward long-term, preventive disease management, thinking beyond the host plant’s growing season in order to hinder pathogens at each stage of their life cycle. To that end and using *P. viticola* as a representative example, we have proposed interventions leveraging genetic, chemical, biological, and physical methods that would expand upon the scope and efficacy of IPM in viticulture. Developing and adopting these interventions requires three key steps: 1) Deepen our understanding of the pathogen’s biology, epidemiology, and ecology; 2) Evaluate the proposed interventions for their effectiveness at managing disease, while accounting for any detrimental or indirect effects on crop yield and the environment; 3) Scale up the interventions, being sure to consider how they can be incorporated into existing commercial vineyard management, how to implement and coordinate on larger, regional scales, and any socio-economic constraints that could inhibit their adoption by farmers.

Regarding the biology, epidemiology, and ecology of *P. viticola*, the major outstanding questions relevant to effective preventive disease control include the following: i) characterizing disease progress after harvest; ii) identifying the environmental or host-related factors that trigger sexual reproduction and when it occurs in the field; iii) unraveling the hormonal communication behind sexual reproduction; iv) quantifying the relationship between primary inoculum abundance and primary contamination intensity; v) uncovering relationships with other organisms that affect primary inoculum quantity and viability; vi) working out the triggers and dynamics behind primary inoculum germination; and finally vii) understanding dispersion pathways of primary inoculum from the soil compartment and leaf litter to the grapevines.

To evaluate these interventions for their disease management potential and for their effects on crop yield and the environment, we must develop robust, quantitative methods to monitor *P. viticola* throughout its sexual cycle. These include molecular tools to quantify primary inocula (Si Ammour et al., 2020; Yang et al., 2023; Poeydebat et al., 2025) and bioassays to assess their viability (Poeydebat et al., 2025), in each compartment harboring primary inocula. Additionally, semi-controlled experimental systems, such as mesocosms, could allow the study of primary contamination cycles under standardized conditions, enabling us to evaluate the efficacy of novel preventive strategies at reasonable scales. Critically, we must also account for and study any indirect and unintended consequences of these interventions, including long-term effects on ecosystem functions and biodiversity, as well as any impact on wine production and quality.

Beyond these advances in pathogen biology and in research methodology, the successful implementation of any interventions targeting the sexual cycle of *P. viticola* requires the development of scalable technical solutions. We must therefore address how they can be incorporated into existing vineyard operations, advocate for regional coordination and implementation, and anticipate socio-economic challenges. Regarding the first point, the majority of these proposed interventions build on techniques, equipment, and infrastructure that already exist in viticulture, from the deployment of resistant varieties to foliar applications to the implementation of a physical barrier such as cover crops. In addition, most of these interventions would be enacted between harvest and budburst, a period that is typically less labor-intensive for viticulturists. With the exception of the two more speculative interventions (*i.e.*, interfere with signaling that regulates mating-type behavior and use the false seedbed technique), we believe these proposed interventions–while innovative in this context–are generally straightforward to integrate into existing viticultural management. Secondly, the success of preventive measures depends on regional, coordinated deployment and the design of pest-suppressive landscapes (Jacquet et al., 2022). Because this pathogen can rapidly propagate via repeated asexual cycles once an epidemic is established, aerially dispersing and colonizing fields in the surrounding area (Aylor et al., 1982; Jacquet et al., 2022), any small-scale preventive management risks being rendered ineffective. Transitioning towards the necessary collective management framework requires robust institutional support and policy initiatives, including educating and supporting winegrowers and fostering regional coordination. Thirdly, socio-economic constraints that may hinder the adoption of new disease management strategies by the winegrowers, particularly those that operate on smaller scales, must be anticipated and addressed. While these proposed interventions would ultimately simplify the winegrowers’ disease management strategy and reduce yield loss from GDM, short-term challenges include the time and labor to learn new techniques, and the potential need for new machinery, chemicals, and biocontrol solutions. Therefore, research into spatial organization, social networks, and collective action is equally crucial in order to support the transition toward the diversified disease management strategies that we believe will enable preventive, long-term and sustainable disease management.

## Data Availability

The original contributions presented in the study are included in the article/supplementary material. Further inquiries can be directed to the corresponding author.

## References

[B1] Abad J. Hermoso de Mendoza I. Marín D. Orcaray L. Gonzaga Santesteban L. (2021). Cover crops in viticulture. A systemic review (2): Implications on vineyard agronomic performance. Oeno. One 55, 1–27. doi: 10.20870/oeno-one.2021.55.2.4481

[B2] Ariyoshi S. Imazu Y. Ohguri R. Katsuta R. Yajima A. Shibata T. . (2021). Identification of biosynthetic intermediates for the mating hormone α2 of the plant pathogen Phytophthora. Biosci. Biotechnol. Biochem. 85, 1802–1808. doi: 10.1093/bbb/zbab098. PMID: 34057177

[B3] Asinari F. Bellotti G. Fedele G. Puglisi E. Caffi T. (2026). Soil management modulates vineyard airborne fungal communities and impacts fungal disease pressure. Front. Plant Sci. 17. doi: 10.3389/fpls.2026.1770877. PMID: 42040271 PMC13106304

[B4] Aylor D. E. Taylor G. S. Raynor G. S. (1982). Long-range transport of tobacco blue mold spores. Agric. Meteorol. 27, 217–232. doi: 10.1016/0002-1571(82)90007-3

[B5] Babadoost M. Pavon C. (2013). Survival of oospores of Phytophthora Capsici in soil. Plant Dis. 97, 1478–1483. doi: 10.1094/PDIS-12-12-1123-RE. PMID: 30708464

[B6] Baker K. F. Cook R. J. (1974). Biological Control of Plant Pathogens. (San Francisco, CA, USA: CABI Databases). doi: 10.2307/3758248

[B7] Bebber D. P. Gurr S. J. (2014). Crop-destroying fungal and oomycete pathogens challenge food security. Fungal Genet. Biol. 74, 62–64. doi: 10.1016/j.fgb.2014.10.012. PMID: 25459533

[B8] Boualleg N. J. Salomon M. V. Vilardell P. Aramburu B. Cabrefiga J. (2024). Control of apple scab in commercial orchards through primary inoculum management. Agriculture 14, 2125. doi: 10.3390/agriculture14122125. PMID: 30654563

[B9] Burruano S. (2000). The life-cycle of Plasmopara viticola, cause of downy mildew of vine. Mycologist 14, 179–182. doi: 10.1016/S0269-915X(00)80040-3

[B10] Carisse O. (2016). Development of grape downy mildew (Plasmopara viticola) under northern viticulture conditions: influence of fall disease incidence. Eur. J. Plant Pathol. 144, 773–783. doi: 10.1007/s10658-015-0748-y. PMID: 30311153

[B11] Carisse O. Rolland D. (2004). Effect of timing of application of the biological control agent Microsphaeropsis ochracea on the production and ejection pattern of ascospores by Venturia inaequalis. Biol. Control. 94, 1305–1314. doi: 10.1094/phyto.2004.94.12.1305. PMID: 18943700

[B12] Clément J. A. J. Magalon H. Pellé R. Marquer B. Andrivon D. (2010). Alteration of pathogenicity-linked life-history traits by resistance of its host Solanum tuberosum impacts sexual reproduction of the plant pathogenic oomycete Phytophthora infestans. J. Evol. Biol. 23, 2668–2676. doi: 10.1111/j.1420-9101.2010.02150.x. PMID: 20964786

[B13] Corkley I. Fraaije B. Hawkins N. (2022). Fungicide resistance management: Maximizing the effective life of plant protection products. Plant Pathol. 71, 150–169. doi: 10.1111/ppa.13467. PMID: 40046247

[B14] Courchinoux E. Roux P.-A. Rodriguez M. Demeaux I. Dupin S. Deliere L. . (2025). Le compostage des feuilles de vignes pour réduire l’inoculum primaire de Plasmopara viticola au vignoble? Ives. Tech. Reviews. Vine. Wine. doi: 10.20870/IVES-TR.2025.9359

[B15] Delbac L. Delière L. Schneider C. Delmotte F. (2019). Evidence for sexual reproduction and fertile oospore production by Plasmopara viticola on the leaves of partially resistant grapevine cultivars. Acta Hortic. 1248, 607–619. doi: 10.17660/ActaHortic.2019.1248.82

[B16] De Vrije T. Antoine N. Buitlelaar R. M. Bruckner S. Dissevelt M. Durand A. . (2001). The fungal biocontrol agent Coniothyrium minitans: production by solid-state fermentation, application and marketing. Appl. Microbiol. Microtechnol. 56, 58–68. doi: 10.1007/s002530100678. PMID: 11499948

[B17] Didelot F. Caffier V. Orain G. Lemarquand A. Parisi L. (2016). Sustainable management of scab control through the integration of apple resistant cultivars in a low-fungicide input system. Agric. Ecosyst. Environ. 217, 41–48. doi: 10.1016/j.agee.2015.10.023. PMID: 38826717

[B18] Dussert Y. Legrand L. Mazet I. D. Couture C. Piron M. C. Serre R. F. . (2020). Identification of the first oomycete mating-type locus sequence in the grapevine downy mildew pathogen, plasmopara viticola. Curr. Biol. 30, 3897–3907. doi: 10.1016/j.cub.2020.07.057. PMID: 32795448 PMC7116238

[B19] Fabritius A. L. Cvitanich C. Judelson H. S. (2002). Stage-specific gene expression during sexual development in Phytophthora infestans. Mol. Microbiol. 45, 1057–1066. doi: 10.1046/j.1365-2958.2002.03073.x. PMID: 12180924

[B20] Fernandes de Oliveira A. F. Serra S. Ligios V. Satta D. Nieddu G. (2021). Assessing the effects of vineyard soil management on downy and powdery mildew development. Horticulturae 7. doi: 10.3390/horticulturae7080209. PMID: 30654563

[B21] Fisher M. C. Henk D. A. Briggs C. J. Brownstein J. S. Madoff L. C. McCraw S. L. . (2012). Emerging fungal threats to animal, plant and ecosystem health. Nature 484, 186–194. doi: 10.1038/nature10947. PMID: 22498624 PMC3821985

[B22] Fontaine M. C. Austerlitz F. Giraud T. Labbé F. Papura D. Richard-Cervera S. . (2013). Genetic signature of a range expansion and leap-frog event after the recent invasion of Europe by the grapevine downy mildew pathogen Plasmopara viticola. Mol. Ecol. 22, 2771–2786. doi: 10.1111/mec.12293. PMID: 23506060

[B23] Fournier P. Pellan L. Jaswa A. Cambon M. C. Chataigner A. Bonnard O. . (2025). Revealing microbial consortia that interfere with grapevine downy mildew through microbiome epidemiology. Environ. Microbiome. 20. doi: 10.1186/s40793-025-00691-9. PMID: 40149015 PMC11948771

[B24] Galbiati C. (1980). Osservazioni su Anteridi, Oognoi ed Oospore di Plasmopara Viticola in foglie. Rivista. Di. Patalogia. Vegetale. 16, 15–17.

[B25] Galbiati C. Longhin G. (1984). Indagini sulla Formazione e sulla Germinazione delle Oospore di Plasmopara viticola. Rivista. Patologia. Vegetale. 20, 66–80.

[B26] Gessler C. Pertot I. Perazzolli M. (2011). Plasmopara viticola: a review of knowledge on downy mildew of grapevine and effective disease management. Phytopathol. Mediterr. 50, 3–44. doi: 10.2307/26458675

[B27] Gessler C. Rumbou A. Gobbin D. Loskill B. Pertot I. Raynal M. (2003). A change in our conception of the life cycle of Plasmopara viticola: oosporic infections versus asexual reproduction in epidemics. Integrated Prot. Production Viticulture 26 (8), 13–16.

[B28] Gobbin D. Rumbou A. Linde C. C. Gessler C. (2006). Population genetic structure of Plasmopara viticola after 125 years of colonization in European vineyards. Mol. Plant Pathol. 7, 519–531. doi: 10.1111/j.1364-3703.2006.00357.x. PMID: 20507466

[B29] Goldammer T. (2018). Grape Grower's Handbook: A Guide to Viticulture for Wine Production (3rd ed.). (Boston, MA, USA: Apex Publishers). Available online at: https://www.wine-grape-growing.com/wine_grape_growing/wine_grape_growing_chapters/ch20_integrated_pest_management_vineyards.htm (Accessed March 20, 2026).

[B30] Harutyunyan S. R. Zhao Z. den Hartog T. Bouwmeester K. Minnaard A. J. Feringa B. L. . (2008). Biologically active Phytophthora mating hormone prepared by catalytic asymmetric total synthesis. Proc. Natl. Acad. Sci. U.S.A. 105, 8507–8512. doi: 10.1073/pnas.0709289105. PMID: 18559862 PMC2438403

[B31] Hasanaliyeva G. Furiosi M. Rossi V. Caffi T. (2024). Cover crops lower the dispersal of grapevine foliar pathogens from the ground and contribute to early-season disease management. Front. Plant Sci. 15. doi: 10.3389/fpls.2024.1498848. PMID: 39588085 PMC11586201

[B32] Heitman J. (2006). Sexual reproduction and the evolution of microbial pathogens. Curr. Biol. 16, 711–725. doi: 10.1016/j.cub.2006.07.064. PMID: 16950098

[B33] Hong C. F. Brewer M. T. Brannen P. M. Scherm H. (2020). Temporal disease dynamics and relative importance of sexual and asexual reproduction of grape downy mildew (Plasmopara viticola) in an isolated vineyard in the North Georgia Mountains, USA. Plant Pathol. 69, 1721–1730. doi: 10.1111/ppa.13263

[B34] Jacquet F. Jeuffroy M. H. Jouan J. le Cadre E. Litrico I. Malausa T. . (2022). Pesticide-free agriculture as a new paradigm for research. Agron. Sustain. Dev. 42. doi: 10.1007/s13593-021-00742-8. PMID: 30311153

[B35] Judelson H. S. Ah-Fong A. M. V. (2019). Exchanges at the plant-oomycete interface that influence disease. Plant Physiol. 179, 1198–1211. doi: 10.1104/pp.18.00979. PMID: 30538168 PMC6446794

[B36] Kassemeyer H. Gadoury D. Wilcox W. (2015). “ Downy mildew,” in Compendium of Grape Diseases, Disorders, and Pests, 2nd ed. Eds. Wilcox W. F. Gubler W. D. Uyemoto J. K. ( The American Phytopathological Society, St. Paul, MN, USA), 46–51. doi: 10.1007/s007090200001

[B37] Kennelly M. M. Gadoury D. M. Wilcox W. F. Magarey P. A. Seem R. C. (2007). Primary infection, lesion productivity, and survival of sporangia in the grapevine downy mildew pathogen Plasmopara viticola. Phytopathology 97, 512–522. doi: 10.1094/PHYTO-97-4-0512. PMID: 18943292

[B38] Ko W.-H. (1978). Heterothallic phytophthora: evidence for hormonal regulation of sexual reproduction. J. Gen. Microbiol. 107, 15–18. doi: 10.1099/00221287-107-1-15. PMID: 27077644

[B39] Ko W.-H. (1988). Hormonal heterothallism and homothallism in phytophthora. Annu. Rev. Phytopathol. 26, 57–73. doi: 10.1146/annurev.py.26.090188.000421. PMID: 41139587

[B40] Koopman T. Linde C. C. Fourie P. H. Mcleod A. (2007). Population genetic structure of Plasmopara viticola in the Western Cape Province of South Africa. Mol. Plant Pathol. 8, 723–736. doi: 10.1111/j.1364-3703.2007.00429.x. PMID: 20507533

[B41] Labarga D. Mairata A. Puelles M. de Toro M. Trochoni J. Pou A. (2024). Organic mulches in grapevine shape bacterial communities in specific vintage and location. Appl. Soil Ecol. 201, 105465. doi: 10.1016/j.apsoil.2024.105465. PMID: 38826717

[B42] Lafon R. Clerjeau M. (1988). Downy mildew. In Compendium of Grape Diseases 72 (11), 938–941.

[B43] Lamour K. H. Hausbeck M. K. (2000). Mefenoxam insensitivity and the sexual stage of phytophthora capsici in michigan cucurbit fields. Phytopathology 90, 396–400. doi: 10.1094/phyto.2000.90.4.396. PMID: 18944590

[B44] Lamour K. H. Hausbeck M. K. (2001). The dynamics of mefenoxam insensitivity in a recombining population of phytophthora capsici characterized with amplified fragment length polymorphism markers. Phytopathology 91, 553–557. doi: 10.1094/phyto.2001.91.6.553. PMID: 18943943

[B45] Li X. Yin L. Ma L. Zhang Y. An Y. Lu J. (2016). Pathogenicity variation and population genetic structure of plasmopara viticola in China. J. Phytopathol. 164, 863–873. doi: 10.1111/jph.12505. PMID: 40046247

[B46] Maddalena G. Russo G. Toffolatti S. L. (2021). The study of the germination dynamics of plasmopara viticola oospores highlights the presence of phenotypic synchrony with the host. Front. Microbiol. 12. doi: 10.3389/fmicb.2021.698586. PMID: 34305864 PMC8297619

[B47] Massi F. Torriani S. F. F. Borghi L. Toffolatti S. L. (2021). Fungicide resistance evolution and detection in plant pathogens: Plasmopara viticola as a case study. Microorganisms 9, 1–18. doi: 10.3390/microorganisms9010119. PMID: 33419171 PMC7825580

[B48] McDonald B. A. Linde C. (2002). Pathogen population genetics, evolutionary potential, and durable resistance. Annu. Rev. Phytopathol. 40, 349–379. doi: 10.1146/annurev.phyto.40.120501.101443. PMID: 12147764

[B49] Muscas E. Cocco A. Mercenaro L. Cabras M. Lentini A. Porqueddu C. . (2017). Effects of vineyard floor cover crops on grapevine vigor, yield, and fruit quality, and the development of the vine mealybug under a Mediterranean climate. Agric. Ecosyst. Environ. 237, 203–212. doi: 10.1016/j.agee.2016.12.035. PMID: 38826717

[B50] Ojika M. Molli S. Kanazawa H. Yajima H. Toda K. Nukada T. (2011). The second Phytophthora mating hormone defines interspecies biosynthetic crosstalk. Nat. Chem. Biol. 7, 591–593. doi: 10.1038/nchembio.617. PMID: 21785427

[B51] Paire C. Dolet M.-A. Hannin H. Samsom A. Olivier V. Chervin C. . (2024). History of breeding programmes for fungus resistant grape varieties in Europe, France and the Occitanie region. Ives. Tech. Reviews. Vine. Wine. doi: 10.20870/ives-tr.2024.8262

[B52] Poeydebat C. Courchinoux E. Mazet I. D. Rodriguez M. Chataigner A. Lelièvre M. . (2025). Digital droplet PCR quantification and field-scale spatial distribution of Plasmopara viticola oospores in vineyard soil. Appl. Environ. Microbiol. 91. doi: 10.1128/aem.01667-25. PMID: 41186406 PMC12724222

[B53] Possamai T. Wiedemann-Merdinoglu S. (2022). Phenotyping for QTL identification: a case study of resistance to Plasmopara viticola and Erysiphe necator in grapevine. Front. Plant Sci. 13, 930954. doi: 10.3389/fpls.2022.930954. PMID: 36035702 PMC9403010

[B54] Prodorutti D. Bugiani R. Philion V. Stensvand A. Coller E. Tosi C. (2024). Irrigation targeted to provoke ejection of ascospores of Venturia inaequalis shortens the season for ascospore release and results in less apple scab. Plant Dis. 108, 1353–1362. doi: 10.1094/PDIS-07-23-1245-RE. PMID: 38035780

[B55] Riemens M. M. van der Weide R. Y. Bleeker P. O. Lotz L. A. P. (2007). Effect of stale seedbed preparations and subsequent weed control in lettuce (cv. Iceboll) on weed densities. Weed. Res. 47, 149–156. doi: 10.1111/j.1365-3180.2007.00554.x. PMID: 40046247

[B56] Rossi V. Caffi T. (2007). Effect of water on germination of Plasmopara viticola oospores. Plant Pathol. 56, 957–966. doi: 10.1111/j.1365-3059.2007.01685.x. PMID: 40046247

[B57] Rumbou A. Gessler C. (2004). Genetic dissection of Plasmopara viticola population from a Greek vineyard in two consecutive years. Eur. J. Plant Pathol. 110, 379–392. doi: 10.1023/B:EJPP.0000021061.38154.22

[B58] Rumbou A. Gessler C. (2007). Greek epidemics of grapevine downy mildew are driven by local oosporic inoculum: a population biology approach. J. Biol. Res. 7, 3–18. Available online at: https://www.researchgate.net/publication/260122975 (Accessed October 12, 2023).

[B59] Salinari F. Giosuè S. Tubiello F. N. Rettori A. Rossi V. Spanna F. . (2006). Downy mildew (Plasmopara viticola) epidemics on grapevine under climate change. Global Change Biol. 12, 1299–1307. doi: 10.1111/j.1365-2486.2006.01175.x. PMID: 40046247

[B60] Santos J. A. Fraga H. Malheiro A. C. Moutinho-Pareira J. Dinis L. T. Correia C. . (2020). A review of potential climate change impacts and adaptation options for European viticulture. Appl. Sci. 10, 3092. doi: 10.3390/app10093092. PMID: 30654563

[B61] Scherer E. E. S. Gisi U. U. G. (2006). Characterization of genotype and mating type in European isolates of Plasmopara viticola. J. Phytopathol. 154, 489–495. doi: 10.1111/j.1439-0434.2006.01136.x. PMID: 40046247

[B62] Schlatter D. Kinkel L. Thomashow L. Weller D. Paulitz T. (2017). Disease suppressive soils: New insights from the soil microbiome. Phytopathology 107, 1284–1297. doi: 10.1094/PHYTO-03-17-0111-RVW. PMID: 28650266

[B63] Si Ammour M. Bove F. Toffolatti S. L. Rossi V. (2020). A real-time PCR assay for the quantification of Plasmopara viticola oospores in grapevine leaves. Front. Plant Sci. 11. doi: 10.3389/fpls.2020.01202. PMID: 32849746 PMC7426466

[B64] Sosnowski M. R. Fletcher J. D. Daly A. M. Rodoni B. C. Viljanen-Rollinson S. L. H. (2009). Techniques for the treatment, removal and disposal of host material during programmes for plant pathogen eradication. Plant Pathol. 58, 621–635. doi: 10.1111/j.1365-3059.2009.02042.x. PMID: 40046247

[B65] Spooren J. Shao Y. Tarrant T. Ploehmacher H. Qi R. Hopkoper S. (2025). Downy mildew disease-suppressive soils transmit a protective core microbiome to the phyllosphere. ISME J. 19, (1). doi: 10.21203/rs.3.rs-6693507/v1 PMC12927876 41670341

[B66] Stark-Urnau M. Seidel M. Kast W. K. Gemmrich A. R. (2000). Studies on the genetic diversity of primary and secondary infections of Plasmopara viticola using RAPD/PCR. Vitis 39, 163–166. Available online at: https://www.researchgate.net/publication/267547769 (Accessed October 12, 2023).

[B67] Steinberg G. Gurr S. J. (2020). Fungi, fungicide discovery and global food security. Fungal Genet. Biol. 144. doi: 10.1016/j.fgb.2020.103476. PMID: 33053432 PMC7755035

[B68] Stewart K. Passey T. Verheecke-Vaessen C. Kevei Z. Xu X. (2025). Venturia inaequalis can initiate sexual reproduction prior to leaf-fall: new electron microscopy evidence. Plant Pathol. 74, 1–8. doi: 10.1111/ppa.70029. PMID: 40046247

[B69] Toffolatti S. L. Russo G. Campia P. Bianco P. A. Borsa P. Coatti M. . (2018). A time-course investigation of resistance to the carboxylic acid amide mandipropamid in field populations of Plasmopara viticola treated with anti-resistance strategies. Pest. Manage. Sci. 74, 2822–2834. doi: 10.1002/ps.5072. PMID: 29749019

[B70] Tomura T. Molli S. Murata R. Ojika M. (2017). Universality of the Phytophthora mating hormones and diversity of their production profile. Nat. Sci. Rep. 7. doi: 10.1038/s41598-017-05380-3. PMID: 28694506 PMC5504046

[B71] Töpfer R. Trapp O. (2022). A cool climate perspective on grapevine breeding: Climate change and sustainability are driving forces for changing varieties in a traditional market. Theor. Appl. Genet. 135, 3947–3960. doi: 10.1007/s00122-022-04077-0. PMID: 35389053 PMC9729149

[B72] Trapp O. Avia K. Eibach R. Töpfer R. (2023). 22nd GiESCO International Meeting. (Ithaca, NY, USA). Available online at: https://hal.inrae.fr/hal-04231667v1 (Accessed October 15, 2025).

[B73] van den Berg M. S. Coops H. Simons J. (2001). Propagule bank buildup of Chara aspera and its significance for colonization of a shallow lake. Hydrobiologia 462, 9–17. doi: 10.1023/A:1013125603555

[B74] Vercesi A. Toffolatti S. L. Zocchi G. Guglielmann R. Ironi L. (2010). A new approach to modelling the dynamics of oospore germination in Plasmopara viticola. Eur. J. Plant Pathol. 128, 113–126. doi: 10.1007/s10658-010-9635-8. PMID: 30311153

[B75] Vercesi A. Tornaghi R. Sant S. Burruano S. Faoro F. (1999). A cytological and ultrastructural study on the maturation and germination of oospores of Plasmopara viticola from overwintering vine leaves. Mycologial. Res. 103, 193–202. doi: 10.1017/S095375629800700X. PMID: 41292463

[B76] Wezel A. Bellon S. Dor T. Francis C. Vallod D. David C. (2009). Agroecology as a science, a movement and a practice: A review. Agron. Sustain. Dev. 29, 503–515. doi: 10.1051/agro/2009004

[B77] Wong F. P. Burr H. N. Wilcox W. F. (2001). Heterothallism in plasmopara viticola. Plant Pathol. 50, 427–432. doi: 10.1046/j.1365-3059.2001.00573.x. PMID: 37945311

[B78] Wyatt T. T. Wösten H. A. B. Dijksterhuis J. (2013). Fungal spores for dispersion in space and time. In Advances in Applied Microbiology 85. (Burlington, MA, USA: Academic Press Inc), 42–91. doi: 10.1016/B978-0-12-407672-3.00002-2 23942148

[B79] Yang L. Chu B. Deng J. Yuan K. Sun Q. Jiang C. . (2023). Use of a real-time PCR method to quantify the primary infection of Plasmopara viticola in commercial vineyards. Phytopathol. Res. 5. doi: 10.1186/s42483-023-00178-w. PMID: 38164791

[B80] Yin L. Zhang Y. Hao Y. Lu J. (2014). Genetic diversity and population structure of Plasmopara viticola in China. Eur. J. Plant Pathol. 140, 365–376. doi: 10.1007/s10658-014-0470-1. PMID: 30311153

